# Progressive multifocal cerebral infarction from intravascular large B cell lymphoma presenting in a man: a case report

**DOI:** 10.1186/1752-1947-5-24

**Published:** 2011-01-20

**Authors:** Pornpong Jitpratoom, Patcharawan Yuckpan, Panitta Sitthinamsuwan, Wattanachai Chotinaiwattarakul, Yingyong Chinthammitr

**Affiliations:** 1 Department of Medicine, Faculty of Medicine, Siriraj Hospital, Mahidol University, Bangkok, Thailand; 2 Department of Pathology, Faculty of Medicine, Siriraj Hospital, Mahidol University, Bangkok, Thailand

## Abstract

**Introduction:**

Intravascular lymphoma is rare, and may present as ischemic stroke. Diagnosis is difficult due to the non-specific presentation and lack of lymphadenopathy, thus leading to frequent instances of autopsy-proven diagnosis. To the best of our knowledge, this is the first report of progressive stroke from intravascular lymphoma diagnosed antemortem by random skin biopsy.

**Case presentation:**

A 42-year-old Thai man presented to our hospital with progressive multifocal cerebral infarction. Despite taking aspirin (300 mg/day), his neurological symptoms worsened. During admission, he developed an unexplained fever and hypoxemia. Magnetic resonance angiography clearly showed patency of all cerebral arteries including the internal carotid and vertebrobasilar arteries. Echocardiography, an antiphospholipid antibody test, cerebrospinal fluid cytology and a bone marrow study were normal. Other laboratory test results showed an elevated lactate dehydrogenase level, nephrotic range proteinuria (3.91 g/day), hypoalbuminemia (1.9 g/dL), a very low high-density lipoprotein level (7 mg/dL) and hypertriglyceridemia (353 mg/dL). Because of suspected vasculitis, pulse methylprednisolone was given with transiently minimal improvement. A random skin biopsy from both thighs revealed intravascular large B cell lymphoma. Chemotherapy was not given due to our patient having ventilator associated pneumonia. He died 10 days after the definite diagnosis was established.

**Conclusion:**

One etiology of stroke is intravascular lymphoma, in which random skin biopsy can be helpful for antemortem diagnosis.

## Introduction

Intravascular lymphoma (IVL) is a rare type of extranodal lymphoma with an aggressive clinical course characterized by proliferation of lymphoma cells within the lumina of vessels, particularly the capillaries, with the exception of large arteries and veins [1,2]. Because of its varied clinical symptoms and the absence of lymphadenopathy, a diagnosis of IVL is extremely difficult to make and many of the reported cases were diagnosed postmortem. We report the case of a 42-year-old man with IVL who presented with the clinical features of progressive multifocal cerebral infarction.

### Case presentation

**Figure 1:**
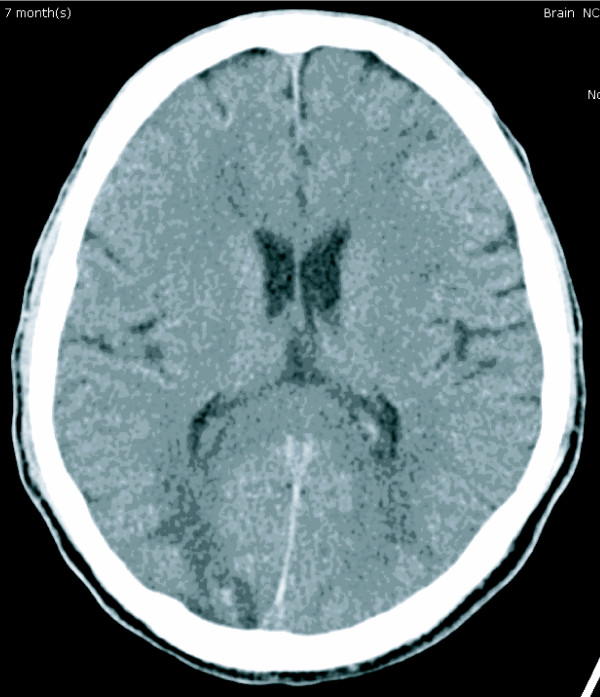
**Computed tomography scan of the brain (with contrast) showing ill-defined hypodensity lesion with multiple hypodensity spots at bilateral parieto-occipito-temporal regions**. The lesion was more on the right than the left cerebral hemisphere.

**Figure 2:**
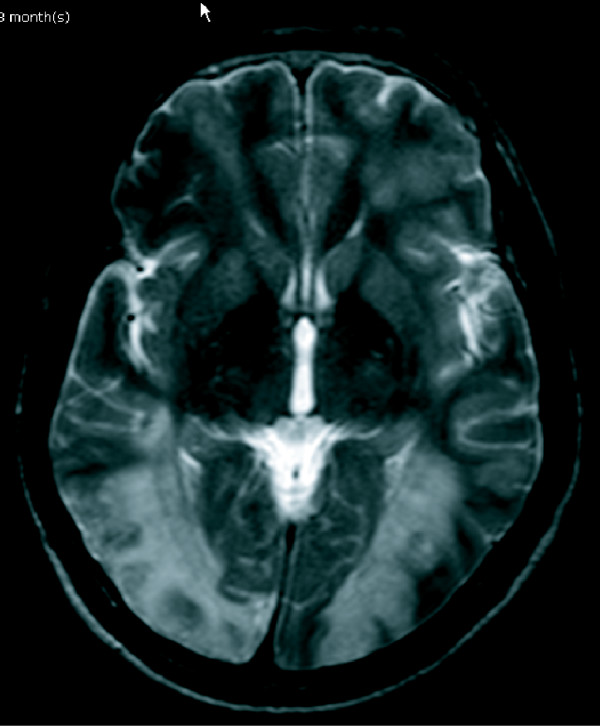
**MRI scan of the brain demonstrating diffuse multi-stage intraparenchymal infarction and hemorrhage at both cerebral and cerebellar hemispheres, initially considered to be vasculitis**.

**Figure 3:**
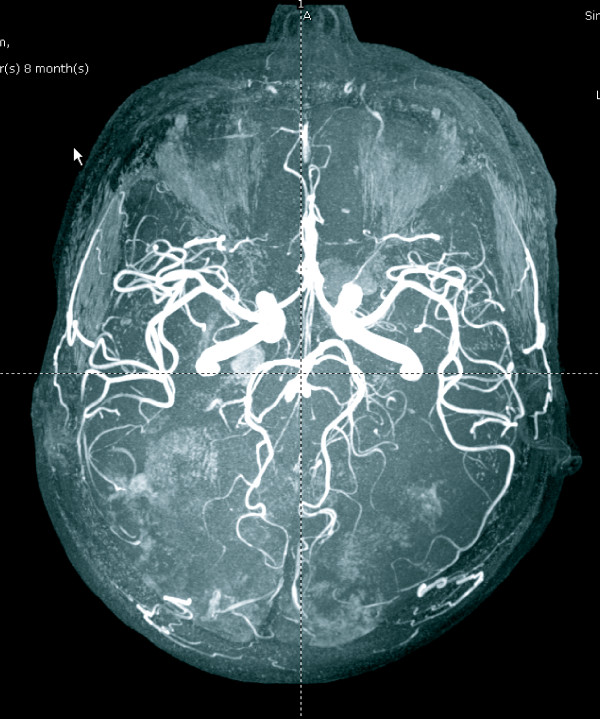
**Magnetic resonance angiogram of the brain demonstrating normal patency of bilateral internal carotid arteries, anterior cerebral arteries, middle cerebral arteries, posterior cerebral arteries, and vertebrobasilar arteries**.

**Figure 4:**
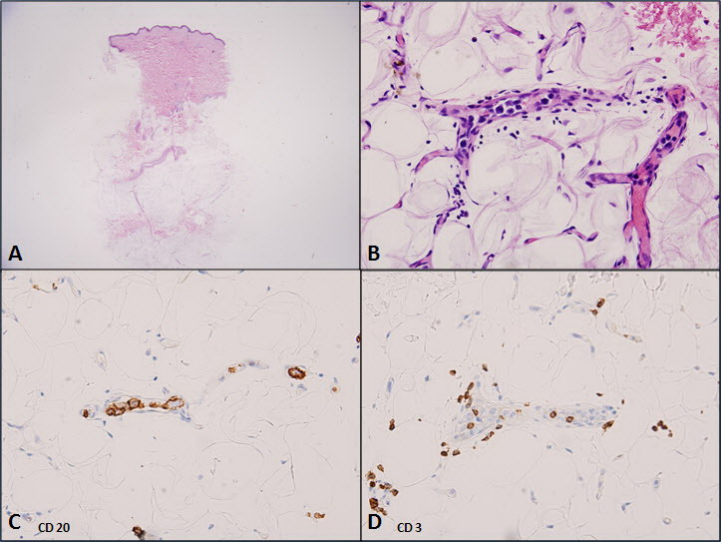
**Skin biopsy**. A) Low magnification showed no apparent diagnostic abnormality (hematoxylin and eosin stain, 40 × magnification). B) High magnification revealed a few large lymphoma cells in the lumens of capillaries in subcutaneous fat (hematoxylin and eosin stain, 400 × magnification). C) Large lymphoma cells marked with CD20. D) Scattered CD3+ small lymphocytes were noted among the large lymphoma cells. Copyright 2005 Blackwell Publishing Ltd. Reproduced with permission.

A 42-year-old previously healthy Thai man developed slow ataxia, motor aphasia and frontal lobe releasing signs, gradually progressing over two months. A computed tomography (CT) scan of the brain showed multiple hypodensity lesions at bilateral occipito-parieto-frontal and cerebellar regions consistent with cerebral infarction (Figure 1). Our patient was treated with aspirin, 300 mg/day. His echocardiography results were unremarkable. A complete blood count (CBC) showed only mild anemia (hemoglobin (Hb) 10.1 g/dL, mean corpuscular volume 80 fL, white blood cell count 6270 cells/mm^3^, platelet count 192,000 cells/mm^3^; neutrophils 72%, lymphocytes 17%, monocytes 11%). Antiphospholipid antibody (lupus anticoagulant, anticardiolipin antibodies, anti-B2GP1 antibodies), antinuclear antibody (ANA), and Venereal Disease Research Laboratory (VDRL) tests were negative. Other laboratory test results showed an erythrocyte sedimentation rate (ESR) of 89 mm/hour, a C-reactive protein (CRP) level of 64 mg/L, and a lactate dehydrogenase (LDH) level of 873 U/L (normal range 225 to 450 U/L). Within two weeks his neurological condition had deteriorated, as noted by developing left hemiplegia and a worsening level of consciousness. Concurrently, he had developed low-grade fever without an identifiable source of infection and an elevated level of LDH (1203 U/L). A repeat CBC still showed only anemia (Hb 8.5 g/dL, WBC count 8220 cells/mm^3^, platelets 160,000 cells/mm^3^). He also had unexplained hypoxemia (PaO_2 _59.5 mmHg, O_2 _saturation 92%, PaCO_2 _35.9 mmHg). Doppler ultrasound scans of the veins of both legs were normal. Intravascular lymphoma was suspected. A bone marrow study showed normal marrow with no evidence of hemophagocytosis and lymphoma involvement on both hematoxylin and eosin and immunohistochemical staining. Because a bone marrow study and cerebrospinal fluid cytology showed unremarkable results, we decided to perform a random skin biopsy on both thighs. A magnetic resonance imaging (MRI) scan of the brain showed diffuse multistage intraparenchymal infarctions and hemorrhages in the bilateral cerebral and cerebellar hemispheres (Figure 2). However, magnetic resonance angiography (MRA) of the brain revealed normal intracranial vasculature (Figure 3). Vasculitis was considered, so our patient was treated with pulse methylprednisolone (1 g/day for five days), with his clinical symptoms partially improving as a result. Interestingly, the pathological findings of the random skin biopsies showed intravascular large B cell lymphoma (IVBL, positive for CD20) (Figure 4). He also had nephrotic range proteinuria (24-hour urine protein was 3.91 g), hypoalbuminemia (1.9 g/dL), hypertriglyceridemia (353 mg/dL) and very low high-density lipoprotein (HDL) (7 mg/dL). We planned to start our patient on chemotherapy but he developed septic shock from ventilator associated pneumonia and died on the 10th day after the definitive diagnosis.

## Discussion

The clinical presentation of IVBL is variable, but the disease has a fulminant course that confers a poor prognosis. Patients may present with a spectrum of symptoms ranging from pyrexia and non-specific constitutional symptoms to multi-organ failure [3,4]. The most common clinical manifestations involve the nervous system and the skin, with at least 68% of patients having involvement of at least one of these organs [4]. Other reported clinical manifestations included fever of unknown origin [4], hypoxemia [5], nephrotic syndrome [6], hemophagocytic syndrome [7], arthritis [8], and myocardial infarction [9]. Anemia and elevated lactate dehydrogenase (LDH) were the most common laboratory findings [3]. The clinical diagnosis of IVL is challenging and often difficult to diagnose antemortem, and the poor prognosis for these patients reflects delays in diagnosis and the institution of chemotherapy. Use of random skin biopsy was reported to help diagnose IVL antemortem [10-12].

Our patient presented with neurological manifestations but did not have cutaneous involvement of IVBL. Initial laboratory abnormalities included mild anemia and elevated LDH, which were non-specific for IVL. Other laboratory test results related to IVL, such as soluble interleukin 2 receptor level [12] and serum ferritin level tests, were not performed. There was no evidence of hemophagocytic syndrome from a bone marrow study. However, histological evidence of IVBL in skin biopsies taken from apparently normal skin has been documented previously. A skin biopsy enabled diagnosis of IVBL in our patient.

The neurological manifestations of IVBL are varied, ranging from focal ischemic events to dementia [3]. Ischemic stroke is a common disease worldwide and is mostly caused by atherosclerotic arterial disease. However, our patient showed some symptoms that guided us to consider IVL as more likely than atherosclerotic arterial disease: our patient had no apparent atherosclerotic risk factor and there was no evidence of a cardiac source of emboli and antiphospholipid syndrome, aspirin did not retard the progression of stroke, he developed an unexplained fever in the second week of hospitalization without an identifiable source of infection and the fever did not respond to empirical antibiotics, he developed unexplained hypoxemia, his serum LDH was elevated to 1203 U/L, he had unexplained nephrotic range proteinuria, hypoalbuminemia and hypertriglyceridemia, and finally, MRA showed normal intracranial arteries.

## Conclusion

Even though intravascular lymphoma is uncommon, the disease should be considered in the differential diagnosis of neurological manifestations or fever of unknown origin with elevated level of lactate dehydrogenase. Our patient's case shows that a random skin biopsy from normal skin can help a physician to diagnose IVBL. We recommend random skin biopsy, a safe bedside procedure, be used in the evaluation of patients in whom the diagnosis of IVL is doubtful, particularly before other invasive investigations are used.

## Consent

Written informed consent was obtained from the patient's next of kin for publication of this case report and any accompanying images. A copy of the written consent is available for review by the Editor-in-Chief of this journal.

## Competing interests

The authors declare that they have no competing interests.

## Authors' contributions

PJ and PY were the primary physicians taking care of our patient and contributed to data collection and drafting of the manuscript. PS, the pathologist consultant, performed the histological examination of skin, revised the manuscript and prepared the figures of pathological findings. WC, the neurologist consultant, analyzed and interpreted the neurological data. YC, the hematologist consultant, analyzed and interpreted the data, and was a major contributor in writing the discussion and revision of the manuscript. All authors read and approved the final manuscript.
